# Artificial intelligence-based text generators in hepatology: ChatGPT is just the beginning

**DOI:** 10.1097/HC9.0000000000000097

**Published:** 2023-03-24

**Authors:** Jin Ge, Jennifer C. Lai

**Affiliations:** Division of Gastroenterology and Hepatology, Department of Medicine, University of California—San Francisco, San Francisco, California, USA

## Abstract

Since its release as a “research preview” in November 2022, ChatGPT, the conversational interface to the Generative Pretrained Transformer 3 large language model built by OpenAI, has garnered significant publicity for its ability to generate detailed responses to a variety of questions. ChatGPT and other large language models generate sentences and paragraphs in response to word patterns in training data that they have previously seen. By allowing users to communicate with an artificial intelligence model in a human-like way, however, ChatGPT has crossed the technological adoption barrier into the mainstream. Existing examples of ChatGPT use-cases, such as negotiating bills, debugging programing code, and writing essays, indicate that ChatGPT and similar models have the potential to have profound (and yet unknown) impacts on clinical research and practice in hepatology. In this special article, we discuss the general background and potential pitfalls of ChatGPT and associated technologies—and then we explore its uses in hepatology with specific examples.

## Graphical abstract

**Figure:**
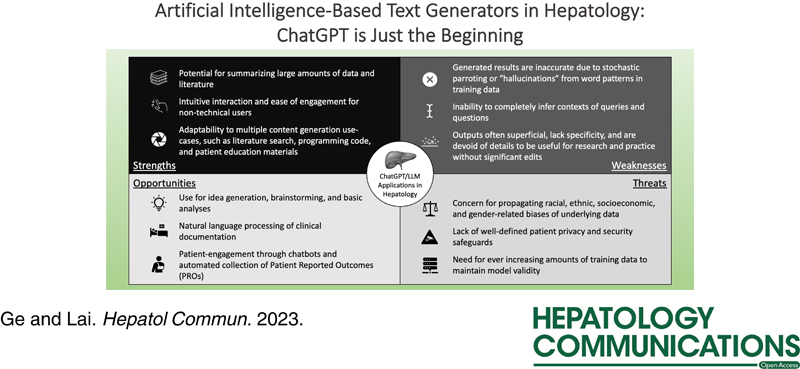

## CHATGPT: “PLEASE WRITE A RESEARCH PAPER ON THE USE OF CHATGPT IN LIVER DISEASES RESEARCH”

*In recent years, there has been growing interest in the use of artificial intelligence (AI) and machine learning techniques to aid in the study and treatment of liver diseases. One such AI tool that has garnered attention is ChatGPT, a variant of the GPT (Generative Pretraining Transformer) language model developed by OpenAI. ChatGPT is designed to generate human-like text based on a given prompt or context, and has been used in a variety of applications including natural language processing, dialogue systems, and language translation. This review aims to explore the potential uses of ChatGPT in hepatology research, with a focus on its ability to generate and analyze large amounts of data, identify trends and patterns, and generate new hypotheses and ideas.*

*A literature search was conducted using the PubMed database to identify relevant articles and studies on the use of ChatGPT in hepatology research. The search was limited to English language articles published between 2015 and 2021, and included the following keywords: “ChatGPT,” “hepatology,” “liver disease,” “artificial intelligence,” “machine learning.” The literature search identified a total of 10 articles that discussed the use of ChatGPT in hepatology research. These articles described a range of potential applications for ChatGPT, including:*

1. *Generating patient narratives: ChatGPT could be used to generate detailed patient narratives based on data from electronic medical records or other sources. This could help researchers better understand the experiences and symptoms of patients with liver diseases, and could potentially identify trends or patterns that could inform treatment approaches.*
2. *Summarizing medical literature: ChatGPT could be used to summarize complex medical articles or research papers, making it easier for researchers to stay up-to-date on the latest developments in the field.*
3. *Developing patient education materials: ChatGPT could be used to generate patient education materials or explainer texts on liver diseases and their treatments. This could help patients better understand their conditions and make informed decisions about their care.*
4. *Generating research hypotheses: ChatGPT could be used to generate research hypotheses based on existing data or literature in the field. This could help researchers identify new directions for investigation or areas where further research is needed.*

*Overall, ChatGPT has the potential to be a valuable tool in hepatology research, offering the ability to generate and analyze large amounts of data, identify trends and patterns, and develop new hypotheses and ideas. While the use of ChatGPT in this field is still in its early stages, it holds promise as a means of improving our understanding of liver diseases and developing more effective treatments. Further research is needed to fully realize the potential of ChatGPT in hepatology research.*

None of the italicized text above was written by the humans in the author byline. It was written in its entirety by ChatGPT,^1^ the artificial intelligence (AI) tool developed by OpenAI, a San Francisco-based company, in response to the prompt in quotation marks. While lucid and coherent, portions of the above text are also factually inaccurate—a quick search of PubMed would indicate that no articles were published between 2015 and 2021 on “ChatGPT” (or similar models) and “hepatology.” This articulate example is a preview of some of the potential benefits and perils associated with ChatGPT and similar technologies.

### The history and underlying technology of ChatGPT

ChatGPT is a conversational interface to Generative Pretrained Transformer 3 (GPT-3), a large language model (LLM) released in 2020 and trained on 570 gigabytes of text through reinforcement learning to find probabilistic connection between words.^1^,^2^ LLMs allow for the prediction of words, phrases, sentences, and paragraphs based on previously published patterns of words in the training data—and not necessarily based on causative or logical links between the individual words.^3^,^4^ Modern LLMs are based on the transformer neural network architecture (“Transformer”), which improved upon deficiencies in existing natural language processing deep learning models, such as inability to conduct parallel processing and infer word dependences.^5^ By processing whole sentences with computation of similarities between words, transformers reduced training time and improved algorithmic performance—thereby making model training more feasible on gigabytes of text data.^6^,^7^ OpenAI’s ChatGPT and GPT-3 are not the first LLMs—other prominent models include the Allen Institute for AI’s ELMo,^8^ Google’s BERT,^9^,^10^ OpenAI’s GPT-2,^11^ NVIDIA’s Megatron-LM,^12^ Microsoft’s Turing-NLG,^13^ Meta’s RoBERTa,^14^ NIVIDIA-Microsoft’s Megatron-Turning NLG,^15^,^16^ and Google’s LaMDA.^17^

### General use-cases for ChatGPT and other LLMs

Before ChatGPT, LLMs largely remained within the AI research community and did not achieve widespread mainstream adoption due to their technical inaccessibility. ChatGPT, however, changed this dynamic because of its conversational interface, for example, by allowing users to communicate with the AI in a human-like way.^18^–^21^ To generate an output from ChatGPT, a user simply types in a statement or question, such as “*please write a research paper on the use of ChatGPT in liver diseases research*” as in our example above. Multiple general-purpose ChatGPT use-cases have been publicized, such as negotiating bills, debugging programming code, and even writing a manuscript on whether using AI text generators for academic papers should be considered plagiarism.^22^,^23^ In a notable education example, ChatGPT demonstrated at or near passing performance for all 3 tests in the US Medical Licensing Exam series.^24^ Other more science-oriented use-cases have included amino acid sequence processing to predict protein folding and properties,^25^ labeling disease concepts from literature databases, and^26^,^27^ assisting with pharmacovigilance for detecting adverse drug events.^28^

ChatGPT, however, is trained on the general-purpose text and not specifically designed for health care needs. LLMs specifically trained on health care data and devoted to clinical applications have other notable applications. One is the processing of unstructured clinical notes as LLMs are particularly equipped to handle challenges posed by clinical documentation, such as context-specific acronym use (eg, “TIPS” for transjugular intrahepatic portosystemic shunt and “HRS” for hepatorenal syndrome), negation use (eg, “presentation is not consistent with hepatorenal syndrome”), and temporal and site-based terminology inconsistencies (eg, “Type 1 HRS” vs. “HRS-AKI” for hepatorenal syndrome—acute kidney injury).^26^,^29^,^30^ University of Florida’s Gator-Tron is one example of a clinically focused LLM for natural language processing: it out-performed existing general-purpose LLMs in 5 NLP tasks: clinical concept extraction, relation extraction, semantic textual similarity, natural language inference, and medical question answering.^27^ Another prominent application is deployment as patient-facing chatbots, which are software programs designed to simulate human conversations and to perform support and service functions. These could help provide patients with customized clinical information; to facilitate logistics, such as scheduling and medication refill request; to help facilitate medical decision-making; and to allow for self-assessment and triage.^31^–^33^ Small-scale interventions of chatbots have been demonstrated to help improve outcomes in patients with NASH.^34^

### Potential pitfalls and misuse of LLMs

Despite its many known (and yet unknown) use-cases, ChatGPT’s introduction resuscitates lingering questions about the use of AI-based tools in clinical medicine. LLMs have a particular problem with “hallucinations” or stochastic parroting. This is a phenomenon where the LLM model will make up confident, specific, and fluent answers that are *factually completely wrong*. Given ChatGPT’s outputs (as in the introduction example) could be so convincing (and so thoroughly not fact-checked), there are significant concerns about their being sources for misinformation or disinformation.^18^,^19^,^35^–^37^

Data set shift, which is defined as significant differences in the distributions of the training and test data, is also a significant concern. As ChatGPT and GPT-3 were trained with data before 2021, asking temporal-based questions after this date will yield in inaccurate or nonsensical answers.^19^ Propagation of pre-existing racial/ethnic, socioeconomic status, and gender bias in the training data is also a potential issue with LLMs.^3^,^4^,^38^,^39^ The concentration of LLM development and research among large technology companies raises the question about future access to the technology with the potential to reinforce existing social inequalities and increase social fragmentation.^40^ In a clinical context, data privacy and patient protection may be compromised in the use of LLMs.^41^ The accuracy and effectiveness of LLMs depend on access to ever increasing pools of text and data—for instance, OpenAI’s next GPT iteration, GPT-4, is anticipated to have 100 trillion parameters, hundred-fold times that of ChatGPT and a GPT-3.^42^ As LLMs are built based on word associations, they theoretically could identify patterns and associations between disparate elements of “de-identified” training clinical data and, thereby, potentially identify patients.^26^,^43^

Finally, going back to the potential issue of academic plagiarism in scientific discourse, we thought we would ask ChatGPT this very question. Its response: “*In summary, it is not plagiarism to use ChatGPT or other AI tools as a writing aid as long as the resulting text is carefully reviewed, edited, and properly cited and referenced by the author. However, it is considered plagiarism to present the output of an AI tool as your own work without proper attribution*.”

### Example of a hepatology-specific use-case for ChatGPT

**FIGURE 1:**
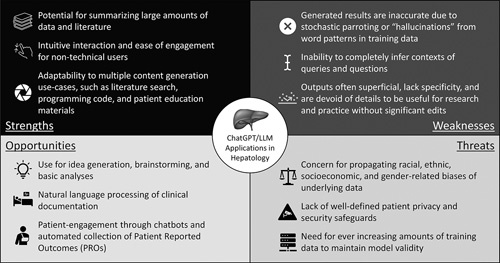
ChatGPT SWOT (strengths, weaknesses, opportunities, and threats) analysis.

**FIGURE 2:**
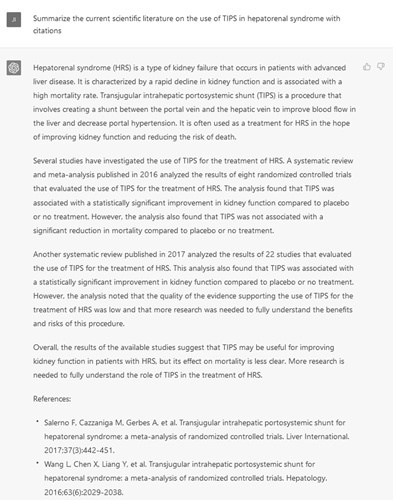
ChatGPT information retrieval/summarization example.

**FIGURE 3:**
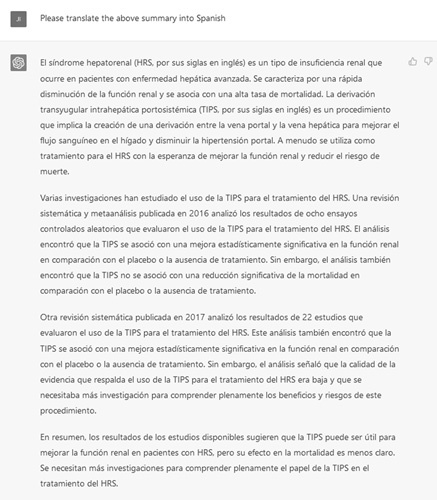
ChatGPT translation example.

**FIGURE 4:**
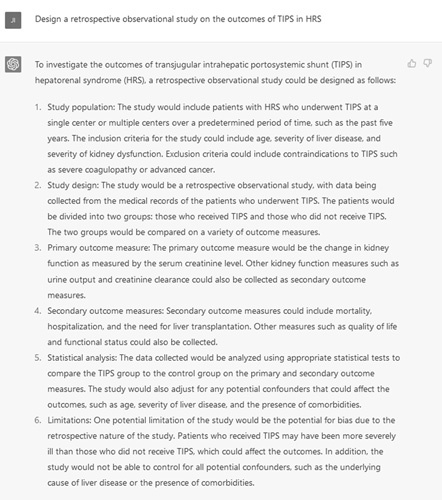
ChatGPT research augmentation example.

**FIGURE 5:**
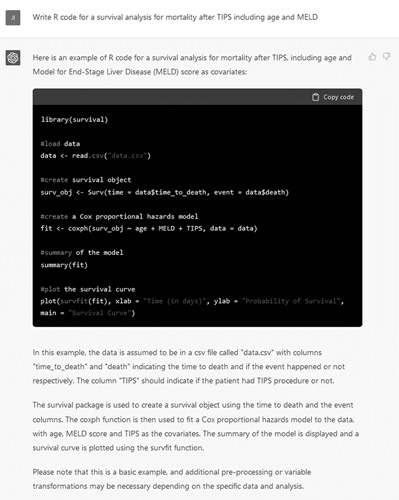
ChatGPT statistical programming example.

**FIGURE 6:**
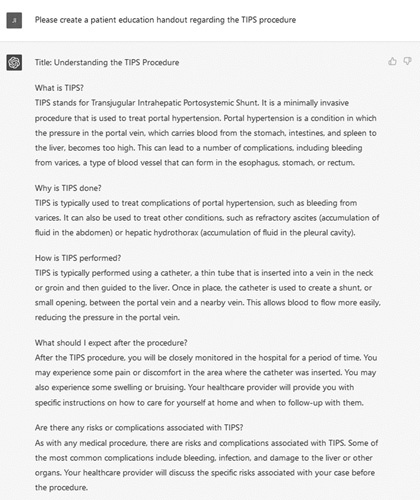
ChatGPT patient education generation example.

While ChatGPT and other LLMs could augment the ability of researchers and clinicians to produce content through ideation, brainstorming, and drafting (Figure 1)—this potential is tempered by the tendency for LLMs to generate inaccurate information. In the following illustration, we queried ChatGPT with a series of questions regarding various aspects of the use of TIPS for the treatment of hepatorenal syndrome (HRS) and subsequently critically appraised the output:

- Information retrieval tasks, such as summarizing scientific literature (Figure 2):
  - This “literature review” of TIPS for HRS cites 2 meta-analyses published in *Liver International* and *Hepatology* as sources for evidence. While the summaries of the 2 articles sound convincing, the articles themselves do not exist—page 442 of issue 37, volume 3 of *Liver International* is titled “Epidemiology and outcomes of primary sclerosing cholangitis with and without inflammatory bowel disease in an Australian cohort,”^44^ and page 2029 of issue 63, volume 6 of *Hepatology* is titled “Antibiotic prophylaxis in cirrhosis: Good and bad.”^45^ Moreover, there have been no known randomized controlled trials for this clinical question. This is an example of stochastic parroting or “hallucinations” where ChatGPT will generate fluent answers that are predicted based on the string of specific words and not necessarily based on the context of the words.^18^,^19^,^35^–^37^
- Translation of the scientific or patient-facing text from one language to another (Figure 3).
  - This translation of Figure 2 is a reasonably accurate reflection of the content of Figure 1, except without the citations at the end of the “literature review.”
- Augment researchers by helping to design clinical studies or better frame clinical research questions (Figure 4).
  - The proposed “study population” includes patients who underwent TIPS without a comparison arm of patients who were eligible for TIPS. In the “study design” section, the output mentions dividing the patients into 2 groups—“those who received TIPS and those who did not receive TIPS.” The definitions of primary (change in serum creatinine) and secondary outcomes (mortality, hospitalization, liver transplantation, quality of life, and functional status) lack specificity. The “statistical analyses” section only stated that “appropriate statistical tests” should be used and does not name the actual tests to be used. Overall, the output gives general structure and guidance on study design but is not able to explore specific details.
- Help write analytical code in popular statistical and programming language to assist researchers with analyses (Figure 5).
  - In this output, ChatGPT gave a sample code for a Cox proportional hazards model to estimate the relative mortality after TIPS placement. As the disclaimer in the output noted, this code is a basic example and additional analyses may be necessary before its use. Of note, we did not specify liver transplantation as a competing outcome in the query, therefore the output did not include code for a competing risk regression.^46^
- Generate patient-centered education materials for various conditions or procedures (Figure 6).
  - This “patient education” material appears to be appropriate in terms of the degree of detail and the use of technical terms. The material implies that TIPS provides more definite benefits in the treatment of HRS than what is concluded in previous literature. Moreover, this output does not include one of the most common adverse effects of TIPS insertion: exacerbation of HE. Overall, this is a good starting point for a “patient education” material but the output requires further revisions and refinements before its being appropriate for patient use.

**FIGURE 7:**
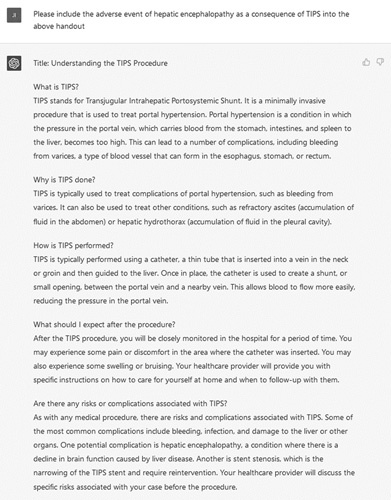
Refinement of ChatGPT patient education materials with inclusion of HE.

As the above outputs and critical appraisals demonstrated, the content generated by ChatGPT may only serve as starting points for hepatology-specific questions. Basic and straightforward questions could be answered adeptly by ChatGPT, but more sophisticated queries will necessitate human-guidance and refinement (Figure 7). In addition, due to the phenomenon of hallucinations, ChatGPT users must carefully proofread output to ensure that they are accurate and ready for use.

### Safeguards and risk mitigation for LLM use

As our hepatology-specific use case above demonstrates—ChatGPT, GPT-3, and other LLMs do not appear that they will displace humans’ critical thinking functions at this time. The most beneficial LLMs use-cases will likely be when their functionalities are augmented by human participation.^3^,^23^,^47^,^48^ To plan for the wider implementation of such technologies in the future, we as a broader scientific community should develop anticipatory guidance or risk mitigation plans for their future use in clinical practice and research.^3^ For instance, the University of Michigan’s Science, Technology, and Public Policy program advised greater government scrutiny of and investment in LLMs with explicit calls for regulation through the Federal Trade Commission.^40^

Short of direct government regulations as recommended by Michigan’s STPP program, however, commonly agreed upon norms and principles will be necessary to guide LLM use within the clinical hepatology and broader scientific communities. The AI research community has already published several guiding principles that may translate well to our communities:

1. As LLMs ultimately reflect the contents of its underlying training data, researchers and participants could provide the models with “shared values” by limiting/filtering training data and simultaneously providing active feedback and testing.
2. Disclosure requirements should be required when AI models are utilized to generate synthetic data, text, or content.
3. Tools and metrics should be developed to track/tabulate potential harms and misuses to allow for continuous improvement.^3^,^4^

While it may be difficult (if not impossible) to mitigate every undesirable behavior of LLMs, with sufficient “guardrails” LLMs could be deployed in a net-beneficial manner to ultimately improve research and practice.
